# Primary hepatic myopericytoma coexisting with multiple cystic hepatic lesions: a case report

**DOI:** 10.1186/s12957-023-02894-1

**Published:** 2023-01-20

**Authors:** Jing Yuan, Jie Li, Zhouhuan Dong, Wei Xu, Zhanbo Wang

**Affiliations:** 1 Department of Pathology, The First Medical Center, Chinese PLA General Hospital, No. 28 Fuxing Road, Haidian District, Beijing, 100853 China; 2 Department of Radiology, The First Medical Center, Chinese PLA General Hospital, Beijing, 100853 China

**Keywords:** Liver tumor, Myopericytoma, Perivascular cell tumor, Hepatic cysts, Immunohistochemistry

## Abstract

**Background:**

Hepatic myopericytoma (MPC) is an extremely rare pathological entity in the liver. Conversely, cystic hepatic lesions are a group of heterogeneous lesions encountered commonly in daily practice. Here, we report a unique case of the coexistence of primary hepatic MPC and multiple cystic hepatic lesions along with our perceptions on its diagnosis and treatment.

**Case presentation:**

A 56-year-old female patient was found to have a left liver mass during a routine physical examination. Computer tomography (CT) and magnetic resonance imaging (MRI) confirmed the existence of a left hepatic neoplasm along with multiple hepatic cysts but could not exclude the possible malignant nature of the neoplasm. Computer tomography (CT) also identified an enlarged mediastinal lymph node with a maximum diameter of 4.3 cm, which further underwent core needle biopsy under CT guidance. A histopathological examination was performed to rule out malignancy. Afterwards, the patient underwent left hemihepatectomy to resect a solid tumor of 5.5 cm × 5 cm × 4.7 cm with multiple cystic lesions which were histopathologically examined to establish the diagnosis of myopericytoma with hepatic cysts. Postoperatively, the patient recovered from the surgery quickly without significant adverse events and was not found to have a reoccurrence of the primary pathological entity.

**Conclusions:**

This is the first reported case of a patient with the co-existence of primary hepatic myopericytoma and multiple cystic hepatic lesions undergoing surgical treatment with eventual recovery.

## Background

Myopericytoma (MPC) is a rare type of perivascular benign neoplasm characterized by spindle cells with contractile features and varied arrangements around the blood vessels [1]. Myopericytoma can be predominantly found in the skin and soft tissues of the lower and upper extremities, and some reported cases have also shown myopericytoma in the intracranial space, nose, urinary tract, and visceral organs [2–8]. Cystic hepatic lesions are a group of heterogeneous lesions with diagnoses ranging from benign to malignant, among which a simple hepatic cyst is the most encountered in imaging studies [9]. Hepatic cysts should be imaged to visualize the number and morphology of lesions and determine whether there is a solid component in the cyst, which is the key imaging feature to differentiate benign ones from malignant cysts [10, 11]. Here, we describe our diagnostic and treatment experience in a case of a 56-year-old female with concomitant hepatic MPC and multiple hepatic cysts, with reference to the differential diagnosis and treatment of choice. It may be of value for clinicians to recognize the presence of this pathological combination for a better management of patient care.

## Case presentation

### Chief complaints

Routine ultrasound found a left hepatic mass with multiple hepatic cysts.

### History of present illness

A 56-year-old female was admitted to our hospital after a mass in the liver was detected by routine ultrasonographic examination a month prior, with a chief complaint of occasional abdominal distension.

### History of past illness

The patient was diagnosed with hypertension at 51 years old and reported no history of hepatitis, tuberculosis, or AIDS.

### Family history

The patient reported no family history of liver malignancy or hepatitis.

### Physical examination upon admission

The patient exhibited stable vital signs, and no issues were detected following a physical examination.

### Laboratory test

Tumor marker tests used can be seen in Table 1.

**Table 1:** Tumor marker test results and normal range

Tumor marker	Results	Normal range
Carcinoembryonic antigen	1.79 µg/L	0–5.0 µg/L
Alpha-fetoprotein	3.58 µg/L	0–20 µg/L
Carbohydrate antigen	29.88 µg/mL	0–37 µg/mL
Cancer antigen-125	8.32 µg/mL	0–35 µg/mL

### Imaging examinations

**Fig. 1:**
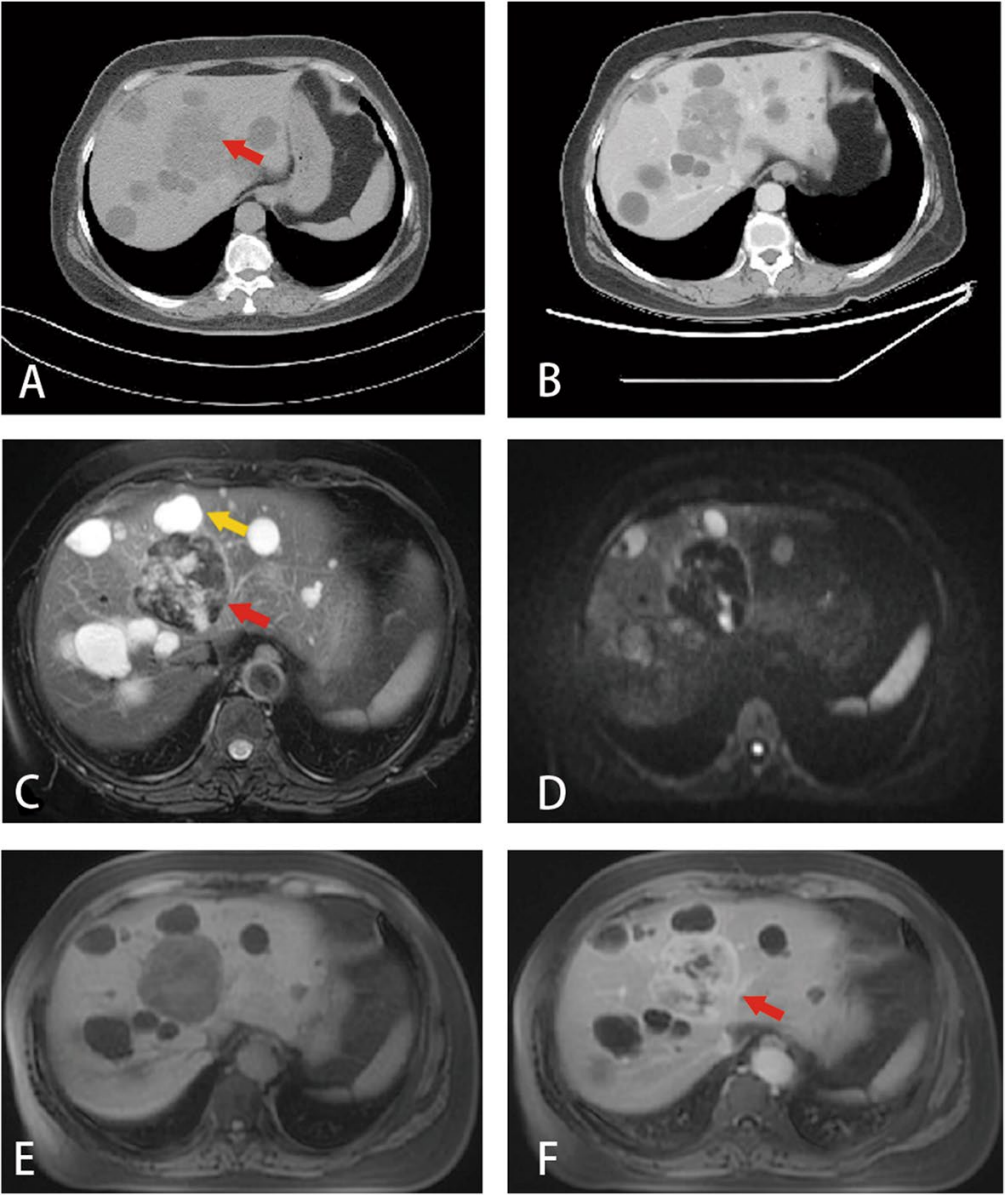
**A** Abdominal CT scan. A slightly low-density mass at the junction of the left and right lobes of the liver (red Arrow). Multiple scattered round water density shadows can be seen, with a clear boundary. **B** CT portal venous phase: the lesion shows progressive heterogeneous enhancement. **C** A preoperative abdominal MRI showed a mass with reduced blood supply in the junction of the left and right lobes of the liver (red arrow), along with multiple hepatic cysts (yellow arrow). **D** The lesion shows heterogeneous hypointensity on DWI, indicating tumor hemorrhage, and the lesion shows no obvious restriction. **E** Abdominal MRI pre-contrast T1WI: the lesion shows heterogeneous hypointensity. **F** MRI shows that the peripheral component of the lesion shows consistent enhancement, and the central component of the lesion shows progressive enhancement in the delayed phase (red arrow). The cystic areas show no enhancement

An abdominal CT scan showed a slightly low-density mass at the junction of the left and right lobes of the liver, existing behind multiple scattered, hypoattenuating, and round lesions with clear boundaries. Portal venous phase CT showed the lesion with progressive heterogeneous enhancement (Fig. 1A, B). An abdominal MRI identified a hypo-vascular mass at the junction of the left and right lobes of the liver with multiple hepatic cysts of various sizes, which could not be ruled out as malignant (Fig. 1C–F). Imaging diagnoses of hemangioma, angiomyolipoma, hemangiosarcoma, and hepatocellular adenoma were considered but dismissed. Moreover, computer tomography (CT) also showed multiple enlarged lymph nodes in the mediastinum, who presented in uniform density without the signs of calcification and necrosis.

### Primary diagnoses

The primary diagnoses are primary hepatic neoplasm and simple hepatic cysts.

### Treatment

The patient underwent a left hemihepatectomy to remove the tumorous lesion in segment 4 of the liver, without any other unique perioperative treatments.

### Outcome and follow-up

#### Gross examination

The hepatic specimen from the resection was 17 cm × 12.5 cm × 7 cm in size and contained a tumorous lesion of the size of 5.5 cm × 5 cm × 4.7 cm. The cut surface of the specimen showed a hard gray-reddish tumorous lesion within the soft gray-yellowish hepatic tissues. Additionally, numerous cystic cavities were observed in the liver, with the largest one being 2 cm × 1.8 cm × 1.5 cm in size with clear fluid inside.

#### H&E and immunohistochemistry staining

**Fig. 2:**
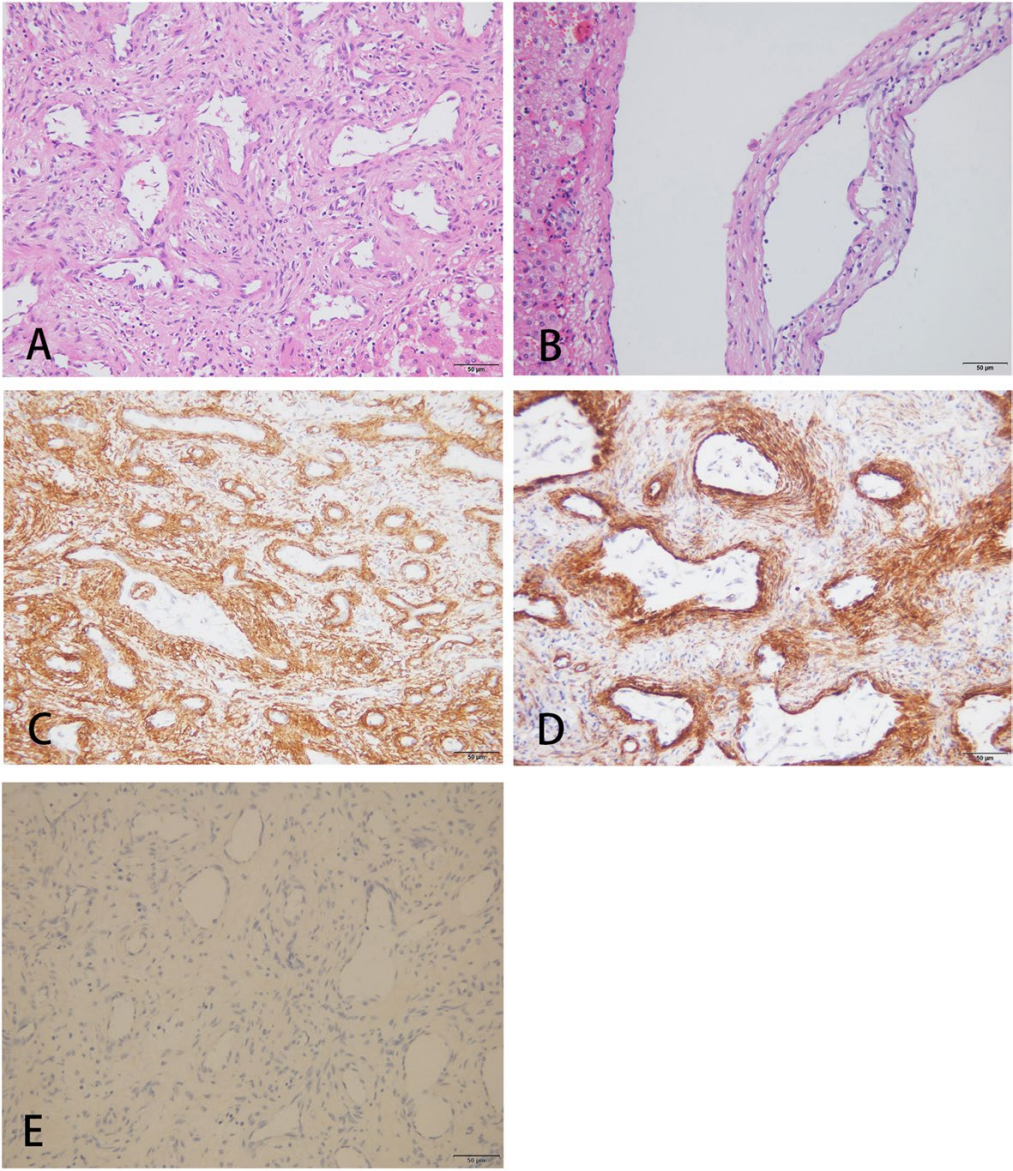
**A** Spindle or epithelioid tumorous cells distributed around the blood vessels in concentric circles (HE staining, × 200). **B** Hepatic cysts lined with single cuboidal epithelium below the liver capsule (HE staining, × 200). **C** Immunohistochemistry showing SMA expression in tumor cells (IHC, × 200). **D** Immunohistochemistry showing H-caldesmon expression in tumor cells (IHC, × 200). **E** In situ hybridization with EBER probe showed spindle tumor cells are negative (× 200)

Routine pathological examination of H&E tissue staining showed that the hepatic tumor cells were oval and spindle and were distributed around the hepatic blood vessels in concentric circles, without degenerative stromal changes, such as fibrosis, hyalinization, or myxoid changes; mitotic figures; tumor necrosis; or vascular invasion being observed (Fig. 2A). The blood vessels were branched, just like those in hemangiopericytoma. Meanwhile, a plurality of cystic cavities lined with single cuboidal epithelium could be seen below the liver capsule (Fig. 2B). Immunohistochemical staining showed that the spindle cells expressed SMA (Fig. 2C) and H-caldesmon (Fig. 2D) but did not detect the expressions of Bcl-2, CD34, S-100, Desmin, HHV-8, CD117, Dog-1, CD21, CD23, CD1a, CgA, Syn, HMB45, and MelenA. The Ki-67 index was low (about 1%). EBER in situ hybridization was performed, and the spindle tumor cells were negative.

#### Molecular examination

Gene + OncoFusion tumor gene-fusion RNA examination with 555 gene fusions revealed the presence of *PDGFRB* mutations but not of *SRF-RELA* gene fusions. Overall, the gene fusion RNA examination did not unveil any common gene mutations or arrangements on the genes, including common mutations in genes related to malignancy or myopericytoma, such as *PRKCSH*, *SEC63*, *LRP5*, *ALG8*, and *SEC61*, or common germline mutations associated with autosomal dominant polycystic liver disease (ADPLD) [12].

#### Follow-up

No recurrence or metastasis was observed, and the enlarged mediastinal lymph node remained unchanged within the subsequent 29 months of follow-up.

## Discussion and conclusions

Requena et al. [13] reported the first case of MPC in 1996 and suggested it was a benign vascular neoplasm likely derived from myopericytes. Two years later, Granter and Kutzner [14, 15] separately reported similar cases and proposed the concept of “perivascular myoma.” It is now well established that MPC is a type of perivascular neoplasm sharing the same lineage with leiomyoma, myofibroma, glomus tumor, and infant angiopericytoma [1]. MPC usually occurs under the skin, often involving proximal as well as distal limbs and neck. Over the past years, sporadic cases of MPCs in the kidney, bronchi, tongue, periampullary tissue, gastrointestinal tract, liver, heart, and brain have also been reported [16], among which hepatic MPC was too rare to be mentioned in the section on digestive tumors of mesenchymal origin by the World Health Organization (WHO) [17]. To the best of our knowledge, there have only been three previously reported cases of primary liver MPC in addition to this one (Table 2) and no literature on the coexistence of hepatic MPC with multiple hepatic cysts. Thus, we believe this report to be the very first one to describe this uniquely occurring condition.

**Table 2:** Clinical features in four cases of hepatic myopericytoma

Case	First authors	Sex⁄age	Site	Presenting symptoms and relevant history	Gross description	Treatment	Follow-up
1	Kang et al. [6]	F/55	3 lesions, segments IV, VI and VIII	Right upper quadrant tenderness	Segment IV and VIII tough and gray nodules without clear capsule; segment VI was hard	IV, VI and VIII segments of the liver resection	Not mentioned
2	Mannanet al. [8]	F/64	Segment IV	Incidentally found during surgery	1.0-cm pale firm nodule	A wedge excisional liver biopsy	Not mentioned
3	Li et al. [18]	F/62	Left-sided liver	Incidentally found during physical examination	6.0 × 5.0 × 4.6 cm, well-circumscribed, solid and gray-white mass	Left lateral segment of the liver resection	No recurrence after 28 months
4	This study	F/56	Segment IV	Incidentally found during routine examination, occasional abdominal distension	5.5 cm × 5 cm × 4.7 cm	Segment of the liver resection	No recurrence after 29 months

Clinically, MPC can develop at any age, but it is most commonly found in middle-aged males. The clinical presentation and histological features of MPC are overall benign although 14 cases of malignant MPC have been reported [19]. Some cases have occurred in AIDS patients and some patients were EBER positive [20, 21].

The diagnosis of MPC and differentiation of benign from malignant MPC are based on morphological and immunological characteristics in histopathological examinations [17]. In our case report, the gross tumor measurement was 5.5 cm in diameter, and the tumor itself displayed classical histological features of MPC, including spindle or epithelioid tumor cells distributed around the blood vessels in concentric circles. Interestingly, other reported histological hallmarks of MPC, including striated muscle differentiation, typical myofibroma, glomus morphology, or mucinous degeneration area, were not seen in this case of hepatic MPC [18]. Additionally, the MPC in our patient did not display an invasive growth pattern, obvious nuclear pleomorphic features, frequent nuclear mitosis, and neoplastic necrosis but did display clear boundaries, no invasion of peripheral liver tissue, no necrosis or mitosis, and a low Ki-67 index (< 1%), all of which did not satisfy the criteria for malignant myopericytoma [19, 22], thereby suggesting the benign nature of the MPC in this patient. The patient was followed postoperatively for 29 months without noted recurrence or metastasis.

Another unique aspect of our case rested in the coexistence of hepatic MPC with multiple hepatic cysts. Hepatic cyst or cystic liver disease includes a heterogeneous group of fluid-filled lesions within the liver parenchyma and can be categorized into simple and complex cysts. Simple hepatic cysts have been defined as lesions with thinly smooth walls lined with bile-like fluid-secreting cuboidal epithelium ranging in size from < 1 to 30 cm in diameter. Furthermore, simple cysts encompass congenital cysts, biliary hamartomas, Caroli disease, and polycystic liver disease (PCLD) [23, 24]. Our patient was imaged via MRI and CT to visualize more than 20 cystic lesions in the different lobes of the liver. Although this satisfies the definition of polycystic liver disease, no history suggestive of familial inheritance was obtained. More significantly, the molecular changes related to PCLD were not observed; therefore, we were only able to give the patient a secondary diagnosis of multiple simple liver cysts.

In the aspect of clinical management, our surgical team could not diagnose the patient as having hepatic MPC preoperatively; thus, they proceeded with the left hepatectomy with the working diagnosis of hepatic malignancy as a precaution. A postoperative pathological exam verified the benign nature of the lesion, and no further treatment was offered to the patient, except for the scheduled follow-up. After 29 months, there was no sign of recurrence or metastasis, further verifying the benign nature of hepatic MPC in our patient. In summary, we presented an extremely rare case of primary hepatic MPC coexisting with hepatic cysts. Our results showed that inpatients with MPC occurring in the deep viscera, which are complicated by other pathological entities such as multiple hepatic cysts, hepatectomy can be the treatment of choice with eventual full recovery.

## Data Availability

The datasets used and/or analyzed during the current study are available from the corresponding author upon reasonable request.

## Abbreviations

MPC: Myopericytoma
CT: Computer tomography
MRI: Magnetic resonance imaging
AIDS: Acquired immune deficiency syndrome
ADPLD: Autosomal dominant polycystic liver disease
PCLD: Polycystic liver disease
PEComa: Perivascular epithelioid cell tumor
GIST: Gastrointestinal stromal tumor
IHC: Immunohistochemical
CK: Cytokeratin
HHV-8: Human herpes virus 8
WHO: World Health Organization
AFP: Alpha-fetoprotein
SMA: Smooth muscle actin
EMA: Epithelial membrane antigen
HEP-1: Hepatocyte paraffin 1
EBER: EBV-encoded RNA
Syn: Synaptophysin
CgA: Chromogranin A
